# European Social Dialogue: History, Characteristics, and Perspectives

**DOI:** 10.1134/S1019331622130147

**Published:** 2022-12-23

**Authors:** L. S. Bisson

**Affiliations:** Institute of Europe, Russian Academy of Sciences, Moscow, Russia

**Keywords:** European Union, social dialogue, social partners, trade unions, labor relations, European Pillar of Social Rights

## Abstract

The author examines the role of the European Social Dialogue (ESD) in decision-making on social policy and labor relations at supranational level in the EU. The author looks into the history and distinctive features of the ESD, its formats, procedures, and legal framework. Based on a review of the institutionalization of social dialogue at the national level in the EU-27, the author draws two conclusions. The first is that the development of social dialogue is uneven across the Union because of the particularities of the social models of the member states and their political and socioeconomic development. The second is that, despite national differences, the coverage of workers by collective agreements in the EU as a whole and the entrenchment of social dialogue at the supranational level make it an integral and distinctive feature of the European social model. An analysis of the evolution of the ESD suggests that there has been a continual move towards a more autonomous status for the social partners. However, because of the 2008–2010 crisis, the ESD’s role has significantly weakened. “A New Start for Social Dialogue” announced by the Juncker’s Commission and several further initiatives are largely declarative. The Court of Justice’s 2021 decision limiting the scope for implementing autonomous agreements at the communitarian level could have a negative impact on the further development of the ESD. Finally, the author positively assesses the possible role of the ESD in overcoming the social consequences of internal and external challenges and the negative effects of transformation of the labor markets.

One of the key elements of “Social Europe” is the European Social Dialogue (ESD). From the very beginning of European integration, the social partners have played an important and, over time, increasingly prominent role in the development of EU social policy. Social dialogue is included in a wide range of instruments which help the institutions of the Union carry out the harmonization of the social sphere, promote the expansion of employment, and guarantee social protection of citizens. In addition to the fact that the European Commission (EC) consults with the social partners before making legislative proposals on a range of issues that regulate social and labor relations, representatives of workers and employers at the supranational level have contributed to the setting of social standards through autonomous agreements. Initiated by the J.-C. Juncker Commission in 2016, A New Start for Social Dialogue^1^ renewed the EU’s commitment to support social dialogue both at the Community level and in the Member States. During the Covid-19 pandemic, which has had a significant impact on the labor market, the institution of social dialogue became an effective tool for maintaining employment in the early stages [ILO, 2020]. The EU Social Summit held in Porto in May 2021 secured an important role for the European social partners in the further development of Social Europe [Bisson, 2021]. In the second half of 2022, the Commission plans to present a plan to strengthen the institution of social dialogue at the community and national levels. Despite the fact that the ESD has led to significant results in the regulation of labor relations in the EU, there are still a number of obstacles to its effective implementation.

The European social dialogue is a complex phenomenon that is widely studied among foreign researchers. The ESD is considered from the point of view of various theoretical approaches. In recent years, the use of social systems theories has been quite widespread: Luhmann’s theory about autopoietic systems [Rogowski, 2000; Hartzén, 2017] and Dunlop’s theory of production relations [Omotayo and Allwell, 2014], according to which social dialogue is a social self-sustaining system with inherent elements such as actors, norms and rules governing the relations of industrial stakeholders, and communication. In addition, the multiplicity of participants and levels of social dialogue in the EU allows researchers to consider it within the framework of the theory of multi-level governance [Keune and Marginson, 2015]. Promising, in our opinion, will be studies of social dialogue within the framework of game theory which allows to establish the asymmetry of the interaction of various actors in the decision-making process [Sørensen et al., 2022].

Among Russian researchers the European social dialogue is most often considered as one of the many components of the EU social policy [Kargalova, 2006; Egorova and Kargalova, 2010; Social Europe in the 21st Century, 2011]. Several scientific articles have been devoted to the development of social dialogue either in individual European countries [Mozhaev, 2001; Polyanskaya, 2017, 2019], or in certain industries [Oleinikova and Murav’eva, 2006; Krysova, 2019]. The role of social dialogue in the regulation of labor relations in the EU is analyzed from the legal point of view in monographs on European labor law [Kashkina, 2009; Egorova, 2018]. However, there is a lack of political science research on the role of social partners in decision-making and social policy development at the supranational level in the European Union.

In the context of the European Union, the term *social dialogue* is used to refer to negotiations between representatives of employers and workers—social partners*—*at various levels: supranational, national, regional, intersectoral, sectoral, and company level. The International Labor Organization (ILO) offers a broader definition of social dialogue, combining it with the notion of tripartism.^2^ The European Social Partners themselves limit the definition of social dialogue to only two-way interaction, even in the case of consultations carried out by the Commission in accordance with the procedure established in Articles 154–155 TFEU. Interaction with EU institutions is not considered by the social partners as part of the ESD. The reason for this distinction is that, in their view, it risks undermining the development of the autonomous nature of the ESD. Within this article, we will also use the typology of A. Bogg and R. Dukes, dividing the ESD into a “guided” social dialogue, initiated and carried out by the Commission, and an “autonomous” one, initiated and carried out by the European social partners themselves [Bogg and Dukes, 2013, p. 468].

The purpose of this article was to identify the role of social partners at the community level and their contribution to deepening the social dimension of integration. The author will rely on a simplified model of the political cycle, first defined by H. Lasswell as a series of stages: agenda setting, policy formulation, decision making, policy implementation, and evaluation [Lasswell, 1956]. The first part of the article will present a general framework for the European social dialogue at the national level in the EU-27 countries. The second part will be devoted to the evolution and main obstacles to the participation of social partners in decision-making at the supranational level, including in an autonomous format. In the final part of the article, conclusions will be drawn and prospects for the development of social dialogue will be discussed.

## SOCIAL DIALOGUE IN THE EU-27 COUNTRIES

Social dialogue has developed at the level of the European Union, reflecting the widespread national practice of the member states. In one form or another, social dialogue takes place in all 27 EU countries, although its significance for industrial relations varies from country to country. It takes various forms, both bilateral and trilateral (or a combination of both), and takes place at both the intersectoral and sectoral levels. Various forms of dialogue reflect the diversity of historically established models of the welfare state in European countries and also correspond to their socioeconomic level of development and political situation. A notable difference is that, in most countries of Western Europe, the current forms of dialogue developed after the Second World War, while in most member states of Central and Eastern Europe, they began to emerge only after the political changes of the late 1980s and early 1990s. Since the 1990s, the development of social dialogue at the EU level has contributed to the development of national bilateral dialogue in some countries where it was previously largely unknown or limited. This is true about the countries that joined the EU in 2004 and 2007. In them, social dialogue, being an integral part of the social *aquis communautaire*, gradually became formalized after joining an integration group [Avdagic, 2002].

The institution of social dialogue is most developed in the EU-15 countries, where cross-sectoral agreements are widespread on a wide range of issues, such as training, employment, health and safety at work, and wages. Despite the occasionally sufficient autonomy of the social partners in these countries, dialogue can be initiated by the government and the agreements reached can be implemented through official state regulations. Public authorities also conduct regular consultations with representatives of trade unions and business in the development of programs and strategies on social and labor issues. In France, for example, the government must consult with the social partners on any legislative or policy proposals relating to individual and collective labor rights, employment, and vocational training. In Austria, Greece, Ireland, Italy, Luxembourg, the Netherlands, Portugal, and Spain, there are tripartite forums on common issues of socioeconomic development. In all these cases, the line between bilateral dialogue and tripartism is blurred. In most new EU member states representatives of employers, trade unions, and government (and sometimes other interest groups) can discuss general economic and social issues. The roles and powers of specialized bodies, usually in a tripartite format, vary greatly, but they usually perform an advisory and deliberative function in relation to draft laws.

Notably, tripartism is perhaps weakest or least visible in Northern Europe. In Denmark, Finland, and Sweden, there is traditionally a clear separation of the areas of competence of the social partners and public authorities. This means that the opportunities for tripartite institutions are limited, and bilateral autonomous dialogue plays a key role. Autonomous intersectoral and sectoral collective is legally binding in this group of countries. However, in recent years there has been some blurring of the dividing line and a growing trend towards trilateral cooperation on specific issues in Denmark and Finland.

If we compare the EU-27 with other regions of the world, then it can be argued that social dialogue is an integral and distinctive feature of the European social model. With all national and regional differences, industrial relations are largely regulated through the negotiations of social partners. Two-thirds of workers in the EU are covered by collective bargaining agreements; in Japan it is one in five workers; in the United States it is one in eight [European Commission, 2012, p. 23]. In 11 EU Member States (Italy, France, Austria, Belgium, Iceland, Sweden, Finland, Denmark, Spain, Portugal, and the Netherlands), collective agreements cover from 99 to 70% of employees.^3^ Despite the fact that trade union membership is declining in all regions of the world, in the EU countries, associations of workers and employers remain quite significant subjects of regulation of industrial relations at the sectoral level.

In the process of forming a single market, there was a constant balancing between economic and social goals. The integration of markets contributed to the transnationalization of industrial relations. While maintaining the key role of collective bargaining at the national level, especially in matters such as wage determination, the EU has gradually promoted social partnership and negotiations at the EU level on political initiatives and allowing the results of agreements to be transferred to the communitarian level. Among other regional organizations where supranational mechanisms of social dialogue are most developed, it is worth mentioning the leading trade block of South America MERCOSUR. Article 20 of the MERCOSUR Social and Labor Declaration includes social dialogue as a fundamental right, stating that the participating states “agree to promote social dialogue at a national and regional level, establishing effective mechanisms of permanent consultation between the representatives of the governments, the employers, and the workers, in order to guarantee, through social consensus, favorable conditions for the sustainable economic growth with social justice in the region and for the improvement of the life conditions of its peoples.”^4^

## HISTORY OF THE ESD DEVELOPMENT

The history of the ESD development is the result of a long political process and can be divided into several periods. The advisory function of European social dialogue was already noted in the 1951 Treaty establishing the European Coal and Steel Community and in the 1957 Treaty of Rome. A significant contribution to ESD development was made by J. Delors. As President of the European Commission in 1985, at a meeting in Val Duchesse, he initiated the involvement of the social partners, represented by the European Trade Union Confederation (ETUC) and two employers' organizations (the Union of Industrialists of the European Community (UNICE) and the European Center for Public Enterprises (CEEP)), in the process of formation of a single market. This event is often referred to as the starting point for ESD development.

The next agreements were the 1991 UNICE, ETUC, and CEEP Joint Agreement, calling for the Commission to have mandatory consultations with the social partners on social relations legislation and to enable them to negotiate autonomously at the Community level. This requirement of the Joint Agreement was included in the Protocol on Social Policy to the Maastricht Treaty of 1992, which meant formal recognition of the role of the social partners in the EU legislative process. The Protocol proclaimed the right of employers and workers acting at Community level to negotiate and enforce binding agreements, either through collective agreements within the member states or through directives adopted by the Council. The ESD received full consolidation in the main text of the Amsterdam Treaty in Articles 137 and 138.^5^ During this period the European Social Dialogue led to the implementation, through Council directives, of three framework agreements (on parental leave in 1996, on part-time work in 1997, and on fixed-term employment in 1999).

In his fundamental book on the history of the ESD, the former Deputy Secretary General of the European Trade Union Confederation Jean Lapeyre notes that in the early years the dialogue between the main players would have stalled if not for the intervention of the Commission [Lapeyre, 2018]. There was little enthusiasm on the part of employers for the development of social dialogue. The intervention of the Commission in these years gave social dialogue the character of tripartism or, in the terminology of A. Bogg and R. Dukes, a “guided” character.

Since 2002, efforts have been made to develop autonomous social dialogue. This was partly done at the initiative of the social partners themselves. At the Social Summit in Laeken in 2001, ETUC, BusinessEurope, and CEEP emphasized the importance of autonomy and insisted on a clear distinction between the different types of communication between the parties: tripartite negotiation, social partners consultations with the Commission and bilateral social dialogue, including both EC-initiated negotiations and negotiations initiated autonomously. Thus, it was an attempt by the social partners to go beyond the “guided” dialogue and take a more independent position. Between 2002 and 2020, six cross-sectoral agreements were concluded (see Table 1), each provided for autonomous implementation by social partners at the national level of the member states, and not by a decision or directive of the Council.

**Table 1:** Table 1

**Cross-sectoral ESD**	
***Agreements implemented in accordance with the EU directive***	***Autonomous agreements***
—Framework Agreement on Parental Leave (Directive 96/34/EC, revised in 2009, Directive 2010/18/EC)	—Framework Agreement on Digitalization (2020)
—Framework Agreement on Fixed-Term Contracts (1999), Directive 1999/70/EC	—Framework Agreement on Active Aging and Intergenerational Approach (2017)
—Framework Agreement on Part-Time Work (1997), Directive 97/81/EC	—Framework Agreement on Inclusive Labor Markets (2010)
	—Framework Agreement on Harassment and Violence at Work (2007)
	—Framework Agreement on Work-Related Stress (2004)
	—Framework Agreement on Telework (2002)

The Lisbon Treaty strengthened the role of the tripartite ESD format. The new Article 152 TFEU established the Tripartite Social Summit on Growth and Employment. The summit was established in 2003 and is held annually between the intersectoral social partners and the President of the Council and the Commission (before the spring meeting of the European Council) and allows the representatives of European business and trade unions to contribute to the EU economic and social strategy for the coming year.

In addition to formal and institutional consolidation, other factors such as the financial and economic crisis of 2008–2009 and the euro area crisis in 2010–2011, also influenced the nature of the ESD. During the postcrisis recovery period social dialogue was weakened by decentralization, a declining scope of regulation for negotiation, and government intervention in wage policy. This period is characterized by a lack of commitment from employers’ associations to enter into negotiations with the European Trade Union Confederation for binding agreements and also by the reluctance of the Commission to submit sectoral agreements of the social partners to the Council for further implementation in the form of decisions or directives. In general, the Commission’s strategy for carrying out structural reforms had an extremely negative impact on the ESD [Degryse, 2017]. According to several authors, during this period national systems for concluding collective agreements on regulating working conditions and wages also suffered noticeably [Dølvik and Martin, 2015]. As expected, the European social partners disagreed on the policy of austerity. While business organizations were generally in favor of the European Commission’s proposals, trade unions at the national and Community level criticized the measures proposed by the EU institutions, which, in their opinion, would lead to unemployment, lower wages, and a reduction in pensions. During this period, the tripartite forums became the only working form of dialogue for the formation of social policy at the EU level.

## CONTRADICTIONS OF THE “NEW START OF SOCIAL DIALOGUE”

A shift in the policy of the European Commission was outlined with the presidency of J.-C. Juncker in the period 2014–2019 [Bisson and Borko, 2019]. In his speech to the European Parliament after being elected to office, Juncker said, “Social dialogue suffered during the crisis years. Now it must be resumed at the national and especially at the European level. I would like to be a President of social dialogue.”^6^ As early as March 2015, the Juncker Commission took action to combat the observed decline in the ESD and announced a New Start for Social Dialogue. Following this, in 2016, the social partners (representatives of employers and trade unions), the Commission, and the President of the EU Council signed a quadripartite agreement of the same name, which confirmed the fundamental role of the European social dialogue in the process of shaping EU social policy, including within the European Semester. The European Pillar of Social Rights (EPSR) 2017 also provides for respect for the autonomy and the right to collective action of social partners and recognizes their right to participate in the development and implementation of employment and social policy, including through collective agreements [Govorova, 2018].

Such a turn was associated, among other things, with a request for “Social Europe” from the citizens of the Union. The problem of trust in supranational institutions and the democratic deficit intensified Eurosceptic sentiments in various EU states. The von der Leyen Commission has repeatedly reaffirmed its commitment to social dialogue in Communications The European Green Deal^7^ and A Strong Europe for Just Transition.^8^ In May 2021 the Porto Social Commitment (signed by the Commission, Parliament, and the European Social Partners) and the Porto Declaration of the European Council highlighted the key role of social dialogue in post-COVID-19 recovery. The EPSR Action Plan presented in March 2021 contains a commitment from the Commission to introduce collective bargaining initiatives for the self-employed in 2021, and a Commission initiative to support social dialogue at the EU and national level is expected before the end of 2022.

A distinctive feature of the postcrisis period in the development of the ESD was a tilt towards “guidence.” As already noted, after the crisis of 2008–2010, without some pressure from the Commission, the employers’ organizations did not show any desire for meaningful interactions with trade unions at the European level [Ebbinghaus and Weishaupt, 2021]. On the other hand, the role and influence of the Commission on the ESD has been labeled as a “shadow of the hierarchy” in a number of research articles. According to this concept, the threat of unfavorable legislation from the Community is an important factor for the European social partners to restore autonomous dialogue and develop norms in an independent mode [Smismans, 2008]. As a rule, the process of coordinating opinions on a draft law already submitted by the Commission takes place with a greater confrontation of the social partners. This ultimately results in the final directive texts being less ambitious than the original proposals [Sørensen et al., 2022].

Thus, the social partners have several tactics for participating in the development or adjustment of social policy in the EU. The path of autonomous intersectoral negotiations largely justifies its effectiveness at stages of the political cycle such as agenda setting and policy evaluation. For example, the 2002 and 2010 autonomous agreements on telework and inclusive labor markets were innovative in their content and proposals. The Parental Leave Agreement, revised in 2009 and adopted as a directive, largely paved the way for the subsequent empowerment of EU citizens with the adoption of the work–life balance directive (EU) 2019/1158.^9^ Thus, the ESD forms the agenda for the further development of the social dimension at the communitarian level. However, the effectiveness of autonomous agreements is reduced due to different practices and procedures for their implementation at the national level. Difficulties remain with the implementation of the provisions of autonomous agreements in some new EU member states where there is often a lack of experience in autonomous negotiations among national labor associations and employers or insufficient coverage of social dialogue. The differentiated effect of the actions of autonomous agreements in the EU as a whole is also associated with differences in national models of industrial relations and legal systems of the member states, as well as with different amounts of changes necessary for their implementation.

The most tangible results of social dialogue at the EU level, in terms of the daily working life of workers and employers, are those agreements that have become legally binding throughout the EU Council directives. This is facilitated by both the ongoing control by the Commission over their implementation and enforcement in the member states and the very status of legal norms to which it is possible to appeal, for example, when protecting the labor rights of workers in those countries where national partners do not have sufficient competence to adopt binding norms. However, in recent years there has been a significant decrease in the interest of the Commission to propose autonomous agreements concluded within the framework of the ESD for consideration by the Council and their further consolidation as directives. In 2018, the Commission rejected a proposal to submit to the Council a 2015 agreement on informing and consulting civil servants and employees of central government administrations. This was the reason for a legal dispute between the European Federation of Trade Unions of Civil Servants (ETUC member), representing the interests of about eight million civil servants in all EU member states, and the European Commission,^10^ as a result of which, on September 2, 2021, the EU Court of Justice issued a resonant decision in favor of the Commission’s right to refuse to allow the European social partners to initiate a legislative procedure at the communitarian level [Dorssemont and Van Malleghem, 2021].

## CONCLUSIONS

Nearly 30 years ago, the Maastricht Treaty established procedures for the European Social Dialogue as part of a broader package of measures to strengthen the social dimension of the European integration. Thanks to the provisions of the TFEU, the European social partners have acquired the competence to become coregulators of industrial relations in the EU. Since the 2000s the social partners have taken a more active and independent stance and have focused on the conclusion of autonomous framework agreements and other “soft law” documents. The autonomy of the ESD implies not only the independence of the social partners in the formation of the agenda but also in its implementation, which, due to the difference in social models in the EU, does not always lead to the expected results. The most tangible effects of social dialogue at the EU level on the daily life of workers and employers are those agreements that have become legally binding across the EU through Council directives.

The “new start for social dialogue” initiated by the Juncker-led Commission is controversial. On the one hand, the supranational institutions of the European Union declaratively support the increased role of social partners which was demonstrated in 2021 both at the Social Summit in Porto and in the Action Plan for the implementation of the European Pillar of Social Rights. On the other hand, despite the fact that many of the Commission’s initiatives are based on the provisions of previously adopted ESD agreements, the Commission avoids the direct participation of social partners in the decision-making process, relying only on their advisory role. In addition, the decision of the EU Court of Justice, which recognizes the right of the Commission to refuse to implement autonomous agreements within the framework of Union legislation, may negatively affect the future of the ESD. Thus, since its inception, the European Social Dialogue has evolved from a relationship of dependency on supranational institutions to a more autonomous position in the 2000s. However, after the crisis of 2008–2010, the role of the ESD has noticeably weakened, it is becoming more and more “guided” with a more prominent role of the European Commission.

At a time when the European Union is on the path of a “double transition,” which will inevitably have side effects on the social sphere and the labor market and is also experiencing the consequences of the COVID-19 pandemic and the armed conflict in Ukraine, social partners can play an important role in maintaining the achieved level of social integration. Representing the interests of employers and trade unions in all sectors and EU member states, thanks to their internal structure and organization, ESD members are able to identify quickly the challenges and interests of both business and workers in response to crisis and transformational phenomena. In addition, the value of social dialogue at the EU level is due to the very process of negotiations and the exchange of views and information, which strengthens internal communication and trust of industrial relations participants.

## Footnotes

1 A New Start for Social Dialogue, Statement of the Presidency of the Council of the European Union, the European Commission, and the European Social Partners, June 27 (2016).

2 ILO Thesaurus, Social dialogue. https://metadata.ilo.org/thesaurus/-1518031573.html.

3 ILO, Statistics on collective bargaining 2018–2019. https://ilostat.ilo.org/topics/collective-bargaining/.

4 Social and Labour Declaration of the Mercosur 2015, p. 16.

5 Articles 154 and 155 as amended by the Treaty of Lisbon.

6 Juncker J.-C. Setting Europe in Motion, Main Messages Opening Statement in the European Parliament Plenary Session, Strasbourg, October 22, 2014. https://ec.europa.eu/commission/presscorner/detail/en/SPEECH_14_567.

7 Communication from the Commission to the European Parliament, the Council, the European Economic and Social Committee, and the Committee of the Regions, *The European Green Deal,* Brussels, Dec. 11 (2019), COM(2019) 640 final.

8 Communication from the Commission to the European Parliament, the Council, the European Economic and Social Committee, and the Committee of the Regions, *A Strong Social Europe For Just Transitions,* Brussels, Jan. 14 (2020), COM(2020) 14 final.

9 Directive (EU) 2019/1158 of the European Parliament and of the Council of June 20, 2019, on work–life balance for parents and carers and repealing Council Directive 2010/18/EU, *Official Journal,* L 188, pp. 79–93.

10 Judgment of the Court (Grand Chamber) of September 2, 2021, Case C‑928/19 P, *European Federation of Public Service Unions (EPSU)* v. *European Commission*.
